# Hospital Expenditure—The Commissariat

**Published:** 1896-11-14

**Authors:** 


### THE INSTITUTIONAL WORKSHOP.

### HOSPITAL EXPENDITURE—THE COMMISSARIAT.

### XXXII.—THE YEARLY AVERAGE COST OF FOOD.

It may now be asked what rate of expenditure on each of the articles enumerated in the uniform system of accounts constitutes a fair average for each class of hospital inmates. It is a question more easily asked than answered. Readers who have followed the series of figures presented week by week under the heading of "Commissariat" cannot fail to have been struck by the wonderful diversity presented under almost every item. It would be easy, but, nevertheless, in many cases disastrous, to fasten in each case on the minimum, and attempt to work the hospital household down to the lowest level. We have attempted to show that special circumstances are often responsible for extremes at either end of these tables, and while never wavering in the conviction that managers owe so much of confidence to the public as to offer explanations of any unusual expenditure, we have from the first declined making any attempt to judge between one and another, in households where all the domestic details differ so widely and are often by the managers themselves so imperfectly understood.

In this matter of hospital commissariat, and, indeed, of hospital management generally, it may be safely asserted that pure theory is worth less than the ink expended in propounding it. All that can be done is to compare, and this, owing to the kind co-operation of many of the best-managed hospitals in the kingdom, it has been possible to do. For the rest, friendly co-operation among those most intimately concerned with the regulation of the hospital food supply should help to remove difficulties and explain anomalies. The matron, for instance, who is perplexed as to whether her household is victualled on the best and most economical system, may without difficulty procure the annual reports of other hospitals of approximate size and circumstances, and may thus ascertain whether her accounts compare favourably or no with those of her neighbours. Mutual inquiries, should discrepancies be revealed, would, it may be believed, be productive of mutual aid, for there are few who would not gladly exchange ideas on the subject engrossing their most earnest attention. The matron at one institution, for instance, finds her bread bill is exactly double per head that of another. What more natural than that she should write inquiring what system is found so economical; is there a fixed allowance for household and patients; what price is paid for bread; and how is waste avoided? Matron No. 2, who has, perhaps, arrived at the conviction that the non-allowance system is by far the most economical, is pleased at the recognition of her success, and glad to explain her methods. Such briefly are the lines on which this comparative system of commissariat might develop. And the managers and secretaries of even the largest institutions might materially aid the cause of good hospital management by studying each other's accounts and laying aside reserve in the discussion of the many knotty points presented by the perpetual variation of cost in the food supply.

It will be convenient to collect together some of the facts already ascertained in a form convenient for general reference. It was found that the yearly cost of boarding the inmates of well-managed institutions was somewhat as follows:—

| | London. | Provincial. |
|---|---|---|
| Doctors ... ... | £50 0 to £60 0 | ... £40 0 to £50 0 |
| Nurses ... ... | 17 0 to 26 0 | ... 13 0 to 18 0 |
| Servants ... ... | 13 0 to 18 0 | ... 13 0 to 16 0 |
| Patients ... ... | 13 0 to 17 0 | ... 10 10 to 14 10 |

No secretarial department can be considered satisfactorily conducted which does not afford the data for ascertaining within narrow limits the cost of each inmate of the hospital. To reckon amounts per bed is, as we have before shown, to disregard entirely the most expensive elements in the household, and is practically no reckoning at all. To reckon per head is still to leave it an open question whether each class of inmates is provided for with due liberality and economy, or whether, owing to extravagance in one department, another is forced to come short. But having settled the expense of boarding doctors, nurses, and patients, it is still very important that those in authority should know the proportional cost of each article which goes to make up the various sums total. In the management of large households, where individual complaint must inevitably be discouraged as much as possible, it will not do to rely on estimates of cost in the aggregate. There may, for example, be a heavy waste in the use of milk, which the housekeeper, aiming at keeping down the weekly total, does her best to atone for by cutting down all fruit and vegetables; or an unnecessary consumption of meat, due to extravagant methods of making beef-tea or wasteful carving, may be visited by extreme parsimony in the supplies for breakfast and supper. It cannot be too often reiterated that the only path to good and economical housekeeping is through an exact knowledge of the quantities used, and a careful comparison with other weeks, other years, and other institutions.

Taking the nurses' diet as a specimen, and noting the average of what has been shown to be the cost in town and country, it may easily be seen how the totals spent on the board of each are made up. The following is the result—not guesswork, but actual facts, so far as it has been possible to ascertain them:—

AVERAGE COST OF BOARDING NURSES.

| | London. | Provincial. |
|---|---|---|
| Meat ... ... | £6 0 to £9 10 | ... £5 10 to £7 0 |
| Fish and Poultry ... | 1 5 to 2 0 | ... 0 15 to 1 0 |
| Milk... ... ... | 1 10 to 2 0 | ... 0 15 to 1 0 |
| Eggs... ... ... | 0 15 to 1 0 | ... 0 5 to 0 10 |
| Butter, Bacon, &c.... | 3 0 to 4 0 | ... 2 10 to 3 0 |
| Bread ... ... | 1 0 to 1 10 | ... 0 18 to 1 5 |
| Vegetables ... ... | 0 15 to 1 5 | ... 0 7 to 0 15 |
| Groceries ... ... | 2 5 to 4 0 | ... 2 0 to 3 0 |
| Malt Liquor ... | 0 10 to 0 15 | ... 0 0 to 0 10 |
| Yearly total ... | £17 0 to £26 0 | £13 0 to £18 0 |

The careful housekeeper will go a step further, and know exactly what quantity of bacon, butter, fish, tea, &c., is used in each department. And it must again be repeated that bills such as the grocer's and butterman's, the butcher's and fishmonger's, must be studied side by side in order to ascertain whether any rise or fall in the one, due to a change of diet, is followed by a corresponding difference in the other.

Variations in numbers must also be closely watched, and in large households this is frequently neglected. To superficial observation the same amount of food is consumed among large numbers when two or three happen to be absent, or when casual visitors are in the house, but if this appears to the housekeeper's eye to hold true, it is a sure sign she has no firm grasp of the reins. An easy-going matron was once heard to lament the extraordinary fatality under which her bills went up rapidly as soon as any additions were made to the household, but refused to show any decrease when some of her party were absent. In family housekeeping where things are managed loosely this phenomenon may very often be noted, but in institution life, where quantities are carefully estimated, and heads are reckoned for the amount of tea to be made and the number of pats of butter needed, the absence of each person should make a perceptible difference in the weekly bills, amounting to at least two-thirds of the average cost per head. That is to say, if the average coast of boarding each nurse works out at 7s. 6d. per week, then 5s. a week should be saved, among large numbers, on each one absent.

